# The Causes of Death in Artificial Tetanus

**Published:** 1882-01

**Authors:** 


### THE CAUSES OF DEATH IN ARTIFICIAL TETANUS.

M. Ch. Richet (*Le Prog. Med.*) gives the result of his experiments in producing death from tetanus due to electrization. The effects produced are analogous to pathological tetanus, and the causes of death probably the same. The causes of death in the artificial condition are of two kinds: *asphyxia*, especially in rabbits, and *high temperature*, in dogs.

Death from asphyxia resulted in about a minute and a half. But asphyxia from simple deprivation of air, does not occur for five minutes. The reason of this difference is in the fact that in electric tetanus, the combustions of oxygen are much more intense in consequence of the access of air into the respiratory tract, so that the carbonic acid accumulates in large quantities in the blood, and death is rapid. In the rabbits the blood does not rise above 40° c.; it varies between 37° to 40°. In the dogs, on the contrary, this temperature rises still higher, and that because, inversely to that which happens in the rabbits, their muscles do not relax, but continue to contract. The respiration is accelerated at the same time with the elevation of temperature.

Death in these animals is due to the excess of heat, for if they are kept, by any cause, in a condition of chill, these accidents do not happen. In the dogs where the temperature rose to 44.5° death was very rapid. When the temperature mounted to 43.7° death sometimes came on very rapidly; at other times death did not occur until after an interval of twenty-four hours. Reasoning from analogy, we would conclude that man, whose temperature is 1° lower than that of the dog, could not endure a temperature of 43.5° and that at 42.7° death would often follow.

Hemorrhages were a grave sign and led to the therapeutic conclusion that bleeding is contra-indicated in the pathological condition. Experiments repeated upon dogs under the influence of chloral, show that the combustions are due, not to the irritation of the nervous system, but rather to the muscular contractions.

M. Richet also thinks that the contractile substance of the muscle is diminished by the oxygen in the blood, since the contraction ceases when the carbolic acid has accumulated.

In regard to tetanus in general M. Richet does not assume that asphyxia and high temperature are the only causes of death, but that nerve exhaustion also plays an important part.—*N. Y. Med. Times.*

